# Measuring the Structure of a Technology System for Directing Technological Transition

**DOI:** 10.1002/gch2.202000073

**Published:** 2020-11-04

**Authors:** Shuanglei Wu, Yongping Wei, Brian Head, Scott Hanna

**Affiliations:** ^1^ School of Earth and Environmental Sciences Faculty of Science the University of Queensland Brisbane 4072 Australia; ^2^ Centre for Policy Futures Faculty of Humanities and Social Sciences the University of Queensland Brisbane 4072 Australia

**Keywords:** ancient Chinese agriculture, complex technology systems, network‐based technology features, technology systems

## Abstract

Technological advancements have generated a “techno‐sphere” within which all humans live. However, the capacity to direct technology development lags far behind technology development itself. This study deciphers the structural characteristics of a technology system using three pairs of features: systemicity and complexity (scalar), centrality and diversity (structural), and adaptability and inertia (structural); and at micro‐, meso‐, and macrolevels. By applying this approach in Chinese agricultural and water technology systems in the Yellow River Region and the Yangtze River Region from the beginning of agriculture in ≈8000 BC to the end of preindustrial agriculture in 1911, it is found that there exist trade‐off relationships between the centrality and diversity of a technology system, there exist alternative dominations of adaptivity and inertia in development of a technology system, and there exist time‐lag phenomena of change in a technology system between mesolevel and macrolevel. It is also identified that a larger‐scale, more diverse and adaptive technology system is observed in the Yellow River Region whereas the technology system in the Yangtze River Region is more rapidly expanding in scale and mainly dominated by inertia. These discoveries will assist increasing the capacity of managing and directing technological transition in future.

## Graphical abstract

It is discovered that there exist trade‐offs between the centrality and diversity of a technology system, and the development alternates between innovative and legacy technologies in time. Time‐lag phenomena and misalignments between the structure and the scale of the systems are identified, as illustrated by an analysis of the development of technology in two river basins in China.

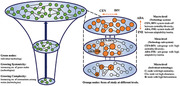

## 1 Introduction

Humankind's amazing innovative capacity has resulted in such tremendous expansion of civilization that our ages are often referred to by their key technological stages—e.g., Stone Age and the Iron Age.^[^ ^1^ ^]^ Globally, technological development has generated a “techno‐sphere” within which humans live.^[^ ^2^ ^]^ The technology‐induced and technology‐magnified unintended and unanticipated disturbances on both human society and the environment have been increasingly evidenced by the unsustainable “locked‐in” development in many parts of the world.^[^ ^3^ ^]^ Our capacity to manage technology development lags far behind technology development itself.^[^ ^4^, ^5^ ^]^

Technology is a complex adaptive system, comprising a number of components and evolving with time.^[^ ^6^, ^7^ ^]^ The development of technology has been most intensively studied in sociology and neoclassical economics. The former, with qualitative approaches, investigates the patterns and trajectories of technological innovations in a co‐evolved societal context;^[^ ^8^, ^9^, ^10^ ^]^ whereas the latter, with quantitative approaches, focuses on cost–benefit analysis using representative metrics of technology such as inputs (e.g., research and design (R&D) investment) and outputs (e.g., number of patents).^[^ ^7^, ^11^ ^]^ However, the internal structure of a technology system and its evolutionary mechanism are neglected. Without opening the “black box” of the system to see what is within, we would not be in the position of managing and directing technology transition.

This study aims to develop a theoretical framework that deciphers the structural characteristics of a technology system in a measurable way. The ancient agricultural and water technology systems in the Yellow River Region and the Yangtze River Region in China are used as case studies to demonstrate the applicability and validity of this framework.

## 2 A Framework for Measuring the Structure of a Technology System

**Figure 1:**
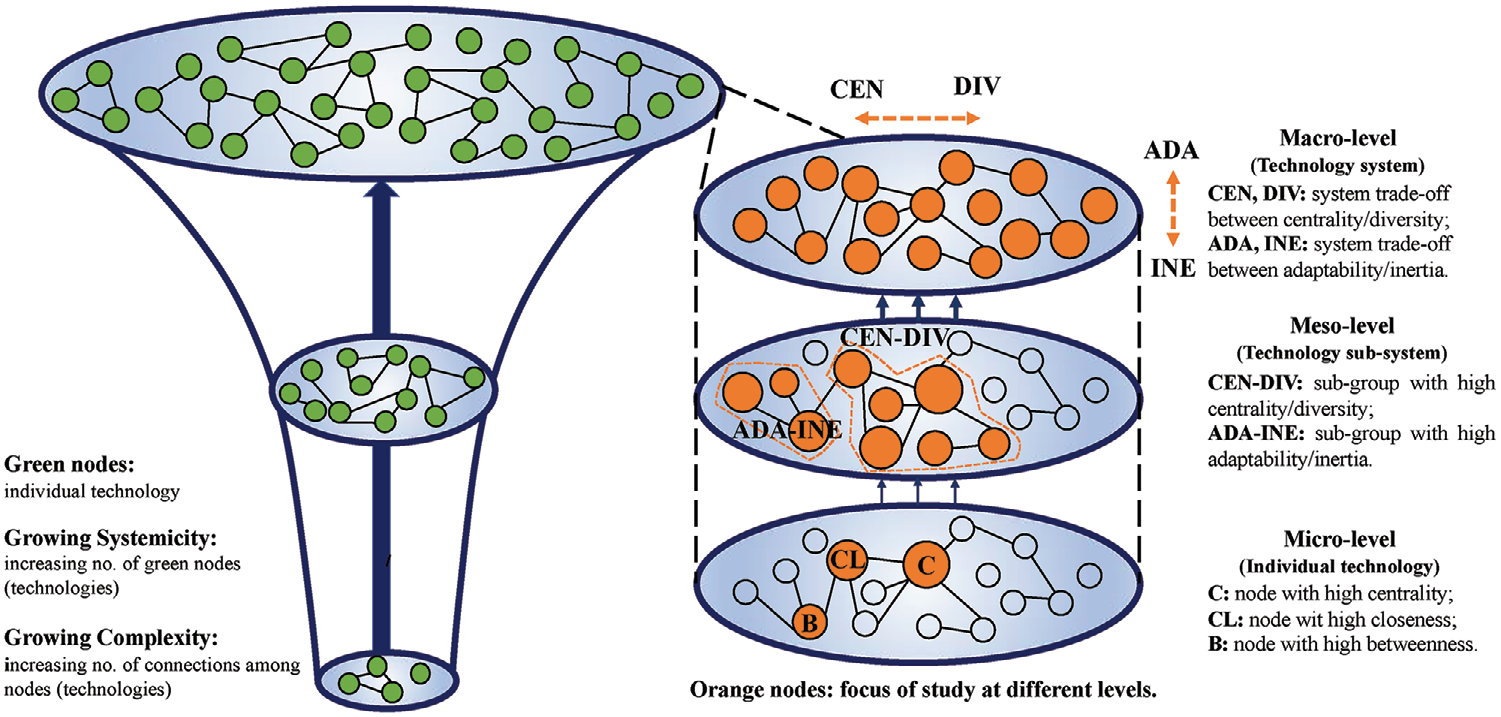
A framework for measuring the structure of a technology system.

We develop a framework for measuring the structure of a technology system based on system theory (**Figure** **1**). As the structure of a complex system is dependent on its scale,^[^ ^12^ ^]^ we first propose a pair of features to measure the scale of a technology system. They are the systemicity (the number of technologies) and complexity (the total connections between technologies). The former indicates how large a system is on a quantitative basis and the latter implies, with more connections, that a technology system could have more interactive influences.

We then measure the internal structure of a technology system at three levels. At the microlevel, we measure how individual technologies connect each other with degree (the number of connections each technology has), closeness (the inverse of total connecting distance for each technology to others), and betweenness (intercategorical connections of each technology) (refer to Section SA in the Supporting Information for calculations). They represent the basic characteristics a node has in a network.^[^ ^13^, ^14^ ^]^

At the mesolevel, we use two pairs of counter‐interacting features to identify the key technological subsystems that occupy important positions in the network and are able to exert greater influences over others,^[^ ^15^ ^]^ thus controlling the directions of technological flow. The first pair is the centrality–diversity of a technology. Centrality measures the intensity of connections as the degree value, reflecting the level of a technological subsystem being propagated within a technology system to facilitate incremental innovation. The diversity represents the extent to which isolated technologies exist within a technology system and is measured by the clustering coefficient, promoting radical innovation.^[^ ^16^ ^]^ Thus, the key technological subsystems should be of high centrality and/or high diversity. The second pair is the inertia–adaptability of a technology system to measure the system behaviors with time. We divide the technology system into two subsystems. The legacy technology subsystem contains technologies inherited from the past, representing the inertia of technological development, and the innovative technology subsystem contains newly developed technologies at the time, representing the adaptability of the overall technology system. Similarly, the key technological subsystems are formed by individual technologies with high inertia and/or high adaptability (more details on how the key technological subsystems are identified can be obtained in Section SA in the Supporting Information).

At a macrolevel, we look at the behaviors of a technology system as a whole. We continue to use the two pairs of previously adopted features at the mesolevel but calculate them based on the average values for all technologies. These two pairs will be examined against the scalar ratio (complexity/systemicity) of a technology system to reveal the relationship between the scale and structure of a technology system.

By understanding the scale and structural features of a technology system, the key technologies that are responsible for driving technological development will be identified. By discouraging or encouraging these technologies, we will have the ability to regulate the scale and structure of the technology system for transitioning toward sustainable development.

## 3 Two Case Studies

**Figure 2:**
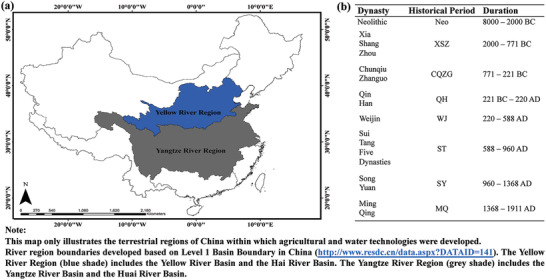
a) The Yellow River Region and the Yangtze River Region in the terrestrial regions of China for agricultural development. b) The temporal ranges of the historical periods in this study.

Agriculture and water technologies have modified our world over a much longer temporal period than any other human innovations. River basins are often recognized as the cradle of ancient civilizations. We apply this framework to examine the evolution of Chinese ancient agricultural and water technology systems in the Yellow River Region and the Yangtze River Region (**Figure** **2**a). These two regions were selected because both were the origins and centers of agricultural development in China, and dryland farming was mainly practiced in the Yellow River Region, whereas paddy field farming dominated in the Yangtze River Region.^[^ ^17^ ^]^ They allow testing if our framework could identify the structural differences of different technology systems within a comparable time scale. Our study period starts from the beginning of agriculture in ≈8000 BC to the end of preindustrial agriculture during the final imperial dynasty in 1911. It included eight historical periods, a timeframe that is long enough to observe the development and transition of a technology system (Figure 2b).

We selected three encyclopedias^[^ ^18^, ^19^, ^20^ ^]^ to provide comprehensive coverage on technological development of agriculture and related socioeconomic conditions in ancient China. We used content‐based text mining methods to extract information required for the framework. We classified these extracted technologies into a hierarchical structure based on their functionalities (refer to ref. ^[^ ^21^ ^]^ for more details), and developed the technology network based on the following principles: a) cumulativeness—all technologies from previous periods were accumulated unless there were specific mentions that they were abandoned;^[^ ^10^ ^]^ b) interdependency—technologies within the same historical period were assumed to be interinfluencing with each other;^[^ ^22^ ^]^ and c) unidirectionality—technology inheritance can only exist from earlier periods to the later one.

## 4 Results

### 4.1 The Scale of the Technology Systems

**Figure 3:**
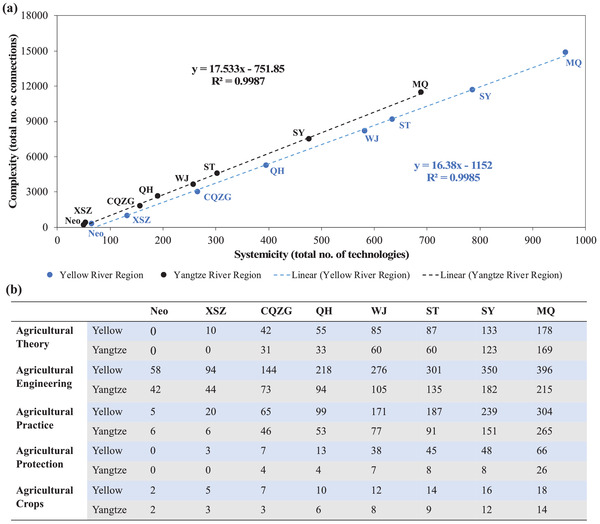
a) Relationships between the systemicity and complexity. b) The number of different types of technologies developed in time in the Yellow River Region and the Yangtze River Region.

The systemicity and complexity of the technology systems presented a highly fitted (*R* ^2^ = 0.99), linearly increasing relationship in both the Yellow River Region and the Yangtze River Region (**Figure** **3**a). The Yellow River Region technology system remained larger in terms of both systemicity (273 more technologies in the final period) and complexities (3390 more connections in the final period) than those in the Yangtze River Region (Figure 3b). There was delay in developing more types of technologies in the Yangtze River Region (refers to Section SA in the Supporting Information for detailed classification). Agricultural theory and agricultural protection technologies appeared in the Yellow River Region during the Xia, Shang, Zhou (XSZ) period (~2000–771 BC); whereas similar technologies did not appear in the Yangtze River Region until the Chunqiu, Zhanguo (CQZG) period (771–221 BC). Agricultural engineering (i.e., tools and infrastructure) always dominated in the Yellow River Region in all periods, but the number of agricultural practice exceeded agricultural engineering in the Yangtze River Region in the Ming Qing (MQ) period (1368–1911 AD).

### 4.2 The Microlevel Features of the Technology System

**Figure 4:**
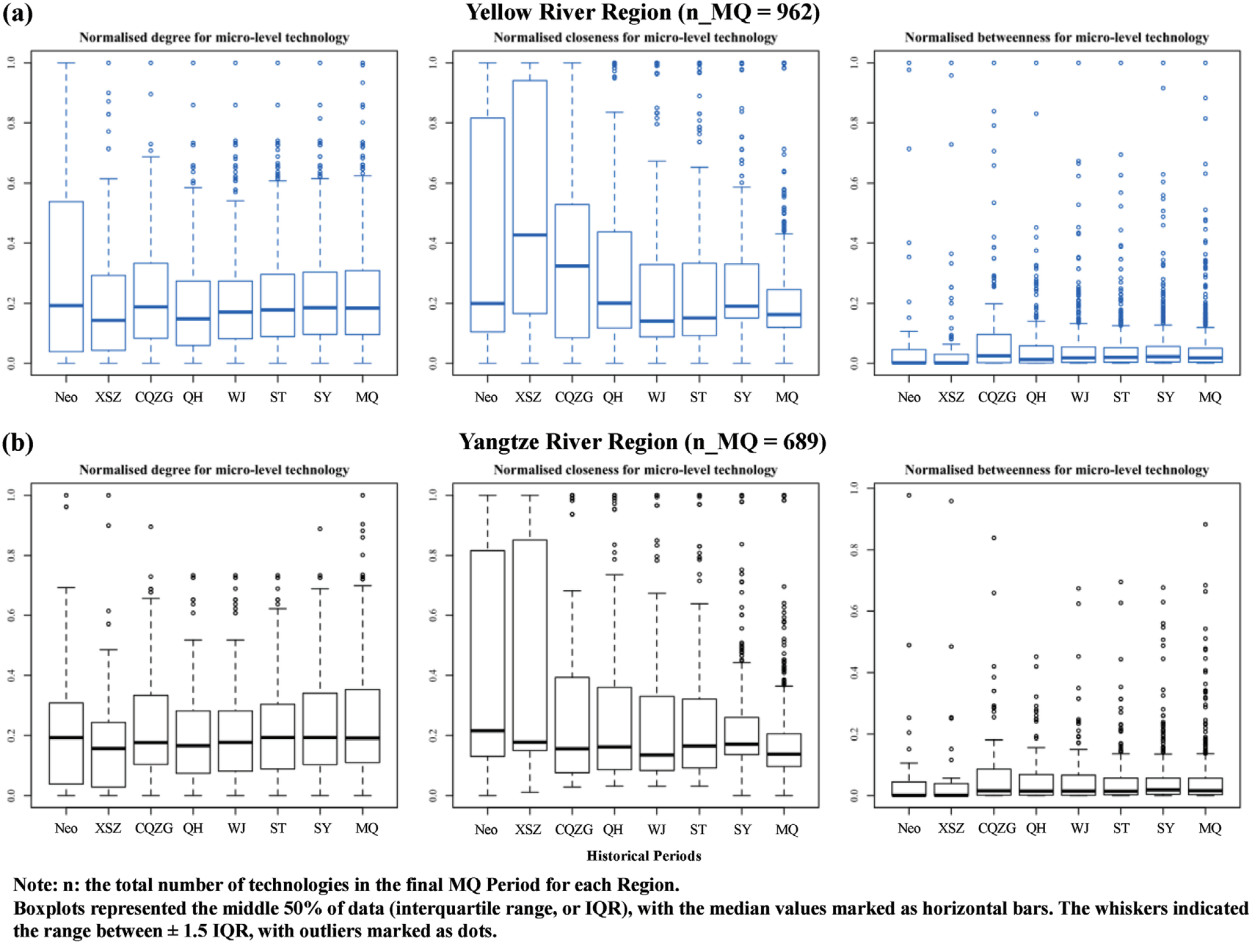
The evolution of microlevel features: degree (left), closeness (middle), and betweenness (right) in a) the Yellow River Region and b) the Yangtze River Region in time.

For both the Yellow River Region and the Yangtze River Region, the average degree and betweenness values of the technology systems were relatively stable in all historical periods (**Figure** **4**a,b). There were greater variations of closeness values during the earlier Neolithic and XSZ periods in the two regions. In another words, more technologies were closely connected during these periods. A general decreasing trend of closeness value for both technology systems was then observed from the XSZ to the MQ period. This indicated that both technology systems rapidly expanded in time, with increasingly less isolated connections (lower closeness) being developed. It was also shown that the range of betweenness values had remained smaller compared to degree and closeness, indicating that there were much fewer technologies with high bridging abilities in both regions for all historical periods. Moreover, slightly greater variations and maximum values of degree and betweenness were observed in the Yellow River Region from the Neolithic to the Song Yuan (SY) period (960–1368 AD). This indicated that the technology system in the Yellow River Region contained individual technologies that were both more connected and with more diversified structural positions, forming a more heterogeneous structure at the microlevel.

### 4.3 The Mesolevel Features of the Technology System

**Figure 5:**
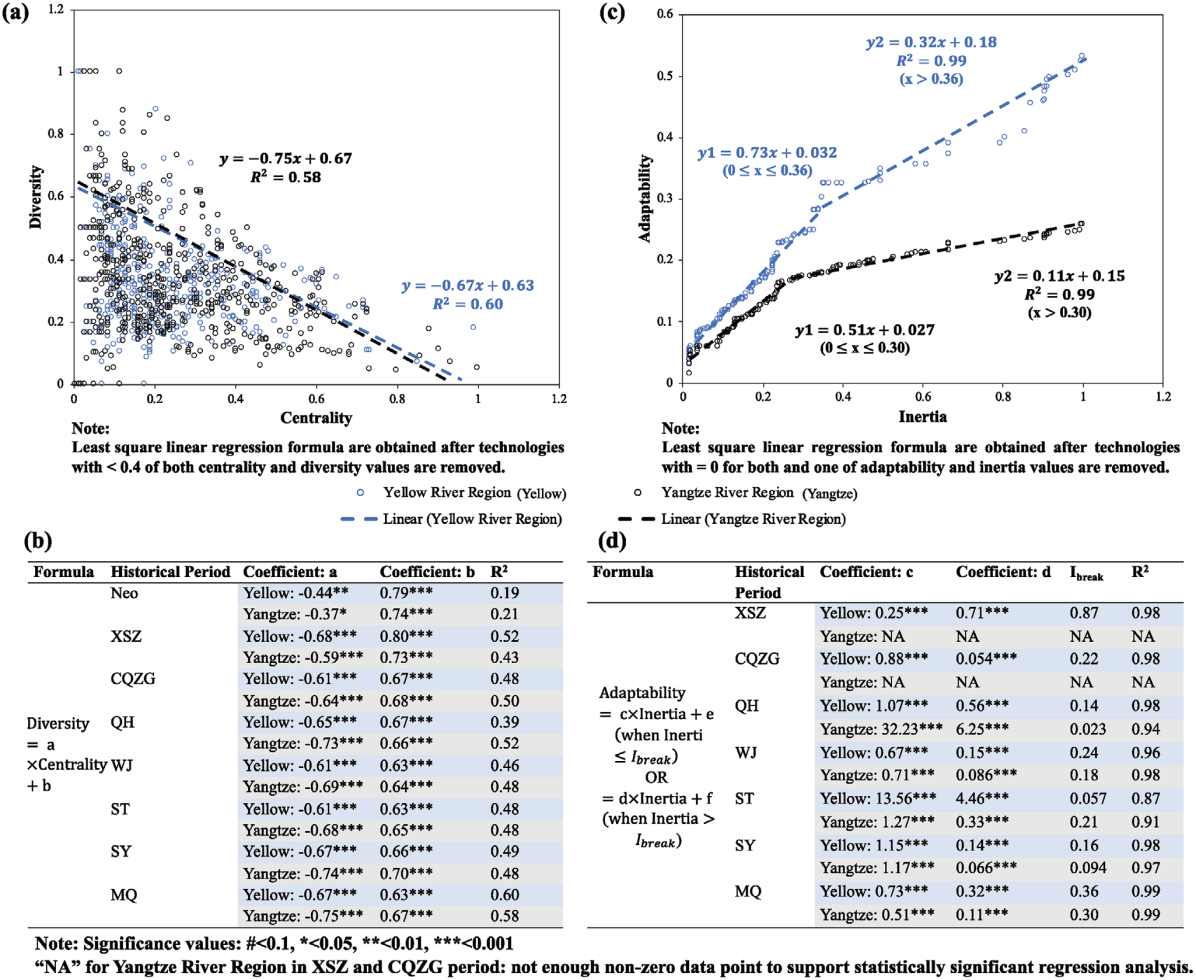
The mesolevel features of the technology system in the Yellow River Region and the Yangtze River Region. a) Centrality–diversity features during the MQ period. b) The linear relationships between centrality–diversity features in time. c) Adaptability–inertia features during the MQ period. d) The linear relationships between adaptability–inertia in time.

At the mesolevel, the key technological subsystem with high centraility and/or diversity in time consisted of about 50–60% of the total number of technologies for both regions. In line with the scalar features, development of agricultural engineering technologies dominated these subsystems in both regions from the Neolithic to Wei Jin (WJ) period (220–588 AD). More technologies related to agricultural practice and agricultural protection appeared in the key subsystems since the Sui Tang, Five Dynasties (ST) period (588–960 AD), especially for the Yangtze River Region (refer to Section SB in the Supporting Information for more details). This indicates that there was greater need for practice and protection technologies for paddy field farming that mainly practiced in the Yangtze River Region. Very similar “trade‐off” relationships between centrality and diversity were also observed for both technological subsystems (**Figure** **5**a). Regression analysis showed that not only were the diversities of the two subsystems linearly reduced with increase in their centralities, but also their rates of changes were similar (ranged between −0.6 and −0.7 except for the Neolithic period; Figure 5b). This indicates a similarly balanced development between the diversities and centralities of the two key technological subsystems, with continuous dominance by the centrality feature (diversity/centrality ratio < 1). However, from the CQZG to the MQ period the key technological subsystem in the Yangtze River Region had greater ratio values and was, thus, more diversified compared to that in the Yellow River Region.

Compared to the key technology subsystems identified by the centrality–diversity features, agricultural theory and agricultural practice technologies had greater adaptability and inertia values in both technology systems toward later periods (refer to Section SC in the Supporting Information for more details). More importantly, both adaptabilities and inertias of the two key technological subsystems linearly increased in time, with similar adaptability/inertia ratios when inertia was <0.35 and bifurcation when inertia was ⪆0.35 in the MQ period (Figure 5c). This curious finding also applies from the WJ to SY periods at inertia values < 0.3 generally (Figure 5d). This indicates both technology systems were homogeneous when their adaptability and inertia values were small and that heterogeneities were observed above the threshold. The key subsystem in the Yellow River Region was demonstrated by distinctively greater increases in adaptability than that in the Yangtze River Region. Moreover, in all periods except the ST period for the Yellow River Region and the Qin Han (QH) period (221 BC–220 AD) for the Yangtze River Region, both subsystems were dominated by the influences of legacy technologies and the inertia feature (average adaptability/inertia ratio < 1) (Figure 5d). This indicated that both key technological subsystems in ancient China relied on development of incremental, legacy technologies inherited from previous periods. It should also be noted that since the SY period, the average ratio was about two times smaller for technologies in the Yangtze River Region than that in the Yellow River Region. Legacy technologies in the Yangtze River Region were, thus, increasingly stronger and more inertial than the key technological subsystem in the Yellow River Region.

### 4.4 The Macrolevel Features of the Technology System

**Figure 6:**
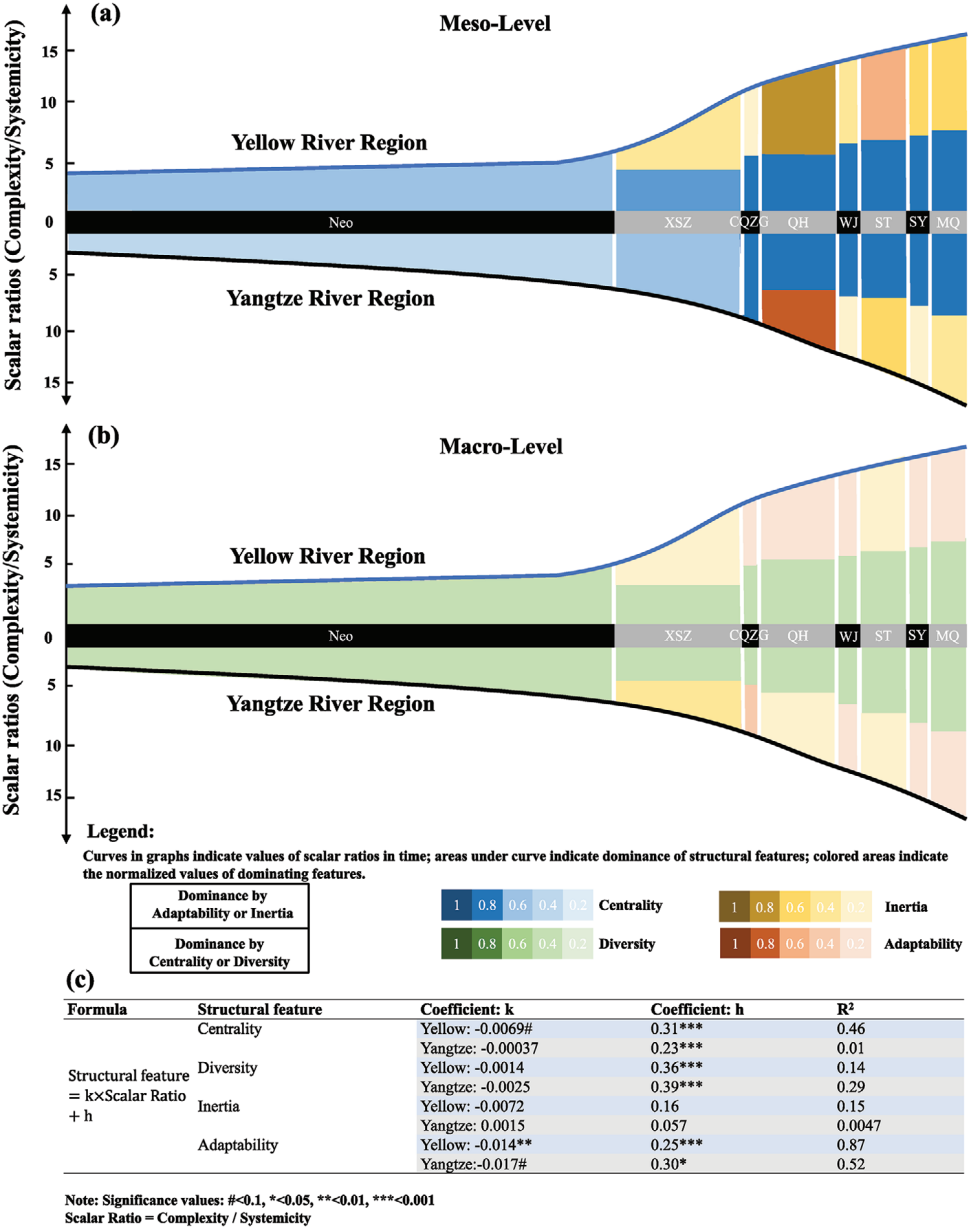
a) The evolution of mesolevel features of the two technology systems. b) Evolution of macrolevel features of the two technology systems. c) Regression relations in time between structural features and scalar features of the two technology systems in the Yellow River Region and the Yangtze River Region at macrolevel.

Both technology systems in the Yellow River Region and the Yangtze River Region showed increasing scalar ratio (represented by complexity/systemicity to reveal the “unit scale” of the technology system) in time, as demonstrated by the increasing boundaries for the two regions in **Figure** **6**a,b. A steeper slope by 7% was observed for the Yangtze River Region, indicating a faster rate of system scale expansion. In terms of structural features at macrolevel (Figure 6c), the centrality of the technology system had been mostly unchanged in the Yangtze River Region (nonsignificant coefficient *k*), whereas a decreasing trend was observed for the system in the Yellow River Region (*R* ^2^ = 0.46). However, the linear reductions with respect to increasing scale in time were not significant for the diversity features for both regions (*R* ^2^ = 0.14 and 0.29). These features indicated that the macrolevel technology system in the Yangtze River Region remained stable in time, whereas the Yellow River Region technology system demonstrated reduced centralization and had stronger potential for more diverse technological development. Conversely, the inertia feature for both regions varied greatly between different historical periods at the macrolevel, with no significant linear relationship with scale identified for either region. Rather, clear decreasing relationships occurred between the adaptability and scale for both regions (coefficient = −0.014 and −0.017, *R* ^2^ = 0.87 and 0.52, respectively). These results demonstrate that with reduced adaptative capacities toward later historical periods, it was also increasingly more difficult to maintain inertia of both technology systems over time (Figure 6b,c).

It was also identified that the structural features of the technology systems at the macrolevel were not consistent with those discovered for key technological subsystems at the mesolevel (Figure 6a,b). Additionally, for both regions, during the periods when the technology subsystems were dominated by adaptability at mesolevel, inertia tended to dominate the overall technology system at the macrolevel. These findings imply a “time‐lagged” effect for technology development in both regions. Even though some key technological subsystems were effective in influencing technological development at the mesolevel, they may not gather sufficient “energy” to modify the overall behaviors of the technology systems at macrolevels. In addition, there appeared to be more adaptability‐dominated periods for the Yellow River Region at the macrolevel, whereas more inertia‐dominated periods were observed for the Yangtze River Region at macrolevel. Therefore, the overall technology system in the Yangtze River Region tended to be more inertial, relying on developing legacy technologies.

## 5 Discussion and Conclusion

We developed a theoretical framework that deciphered the structural features of a technology system in a measurable way and demonstrated its applicability by comparing the evolutions of ancient agricultural and water technology systems in the Yellow River Region and the Yangtze River Region from the beginning of agriculture in ≈8000 BC to the end of imperial China and preindustrial agriculture in 1911.

Our framework, in a measurable way, identified the structural patterns and key differences of the agricultural and water technology systems between the Yellow River Region and the Yangtze River Region. Key findings include the following: 1) both technology systems were characterized by very consistent and slow changes in scale (systemicity/complexity); 2) both systems were less adaptive at the microlevel (high closeness) and highly centralized at the mesolevels (dominated by centrality) during the Neolithic and XSZ periods. 3) A larger‐scale, more heterogeneous (more diverse and adaptative) technology system was observed in the Yellow River Region from the CQZG to ST periods, whereas the technology system in the Yangtze River Region was more rapidly expanding in scale and was mainly dominated by inertia toward the later SY and MQ periods.

Both natural and socioeconomic factors had interactively driven the evolutions of these structural patterns and differences between the technology systems in the two regions. Societal development during the Neolithic and XSZ periods was primitive and highly constrained by climate conditions and availability of natural resources, thus demonstrating high centrality at the micro‐ and mesolevels of the technology systems.^[^ ^23^, ^24^ ^]^ The unification of the nation in 221 BC and a top‐down government structure that prioritized agriculture‐driven technology development (e.g., iron plows and large‐scale irrigation infrastructures) led to a greater scale and more heterogeneous (more diverse and adaptative) structure in the Yellow River Region technology system from the CQZG to ST periods, which contributed to the formation of the agricultural and societal centers in the Yellow River Region.^[^ ^25^, ^26^, ^27^ ^]^ During most of the QH and SY periods in the Yellow River Region, the relative population proportion maintained at about 45–65% of the whole country.^[^ ^28^ ^]^ Cooling climate, frequent wars and invasions, and increasing floods and droughts caused population migrations from the northern to the southern China, transferring advanced agricultural and water technologies to the Yangtze River Region.^[^ ^29^ ^]^ This led to the more rapid expansion in scale and more legacy technologies in the Yangtze River Region technology system toward the later SY and MQ periods, forming a new agricultural center in the Yangtze River Region. The population of this region increased from below 30% to over 45% of the total population.^[^ ^29^ ^]^

The quantitative findings measured with our framework in the case studies consolidate existing empirical and qualitative studies: technological development in ancient China favoring long‐term stability, mainly via incremental innovations,^[^ ^30^, ^31^, ^32^, ^33^ ^]^ trade‐off relationships between centrality and diversity and temporal alternation of adaptivity and inertia,^[^ ^34^, ^35^ ^]^ multiscalar effects of technology reconfigurations,^[^ ^11^, ^36^ ^]^ and combined effects of scale and structure on system transitions.^[^ ^37^, ^38^ ^]^

The proposed framework provides implications in managing and directing transitions of technology systems. First, it enables directing transitions of a technology system through the choices of structural features. Our findings show that there existed “trade‐off” relationships between the centrality and diversity of a technology system and that adaptivity and inertia alternatively dominated development of the technology system at both meso‐ and macrolevels. It implies that technology development decision‐making can seek balance between centralized, incremental innovation for diffusions of existing technologies and diverse, radical innovations for technological transitions, and also balance between adaptability and inertia in the technology system. Second, this framework enables directing transitions of a technology system through identification of the key technological subsystems. Our findings show that the change in structural features of individual technologies and key technological subsystems with large interactive influences at micro‐ and mesolevels occurred earlier than those at macrolevel, i.e., these existed time‐lag phenomena. It implies that technology transitions should target at driving changes in the key technological subsystem at mesolevel to allow sufficient response time windows for change to be effectively and timely propagated to the overall technology system at macrolevel. Finally, this framework enables directing transitions of a technology system through identification of the scalar effect and its interactions with system structure. Our findings show that the relationship between systemicity and complexity of a technology system was positively linear, while the relationships between the structure and scale of a technology system could be significant (adaptability and centrality) or not (diversity and inertia). It implies that the impacts of the varying scales of a technology system on its structural features should be taken into account when the key technological subsystems targeted for transitions.

It should be noted that contemporary technology systems, for example, the green energy consumption and transportation, are generally greater in scale and have more complex structures, of which greater transition momentums are required.^[^ ^37^, ^38^, ^39^ ^]^ This points to future research direction in applying our framework and testing the scalar and structural relationships in modern technology systems. Another future research challenge is to define “a good technology system” based on its impacts on both human society and the environment. In additions, due to coarse and limited data availability, this study might not fully capture the detailed process of technological evolutions and precisely differentiated directions of connections between technologies (i.e., two‐directional, directional, or more influential).

In conclusion, technological development has globally generated a “techno‐sphere” within which humans live. It will tend to move increasingly faster, become more efficient, and generate more unpredictable and potentially unintended impacts. We believe our framework developed and its future improvements will assist policy makers, technology suppliers, and industrial managers to restructure current technology systems and proactively directing, rather than passively reacting to, a more drastically changing human society and natural environment in future.

## Conflict of Interest

The authors declare no conflict of interest.

## Supporting information

Supporting Information

Supplemental Material
