# Supplementary material for: Measuring the Structure of a Technology System for Directing Technological Transition
Source: Glob Chall. 2020 Nov 4;5(2):2000073. doi: 10.1002/gch2.202000073 (PMC7857126; doi:10.1002/gch2.202000073)
Supplement: Supplementary file 1 — Supporting Information [file GCH2-5-2000073-s001.pdf]

© 2020 Wiley-VCH GmbH

Global Challenges

Supporting Information

for *Global Challenges,* DOI: 10.1002/gch2.202000073

Measuring the Structure of a Technology System for Directing Technological Transition

Shuanglei Wu, Yongping Wei, Brian Head, and Scott Hanna*

Measuring the structure of a technology system for directing technological transition

Authors: *Shuanglei, Wu*[1], *Yongping, Wei*[1,*], *Brian, Head*[2], *Scott, Hanna*[1]

Affiliations:

[1]: School of Earth and Environmental Sciences, Faculty of Science, the University of Queensland, Brisbane 4072, Australia.

[2]: Centre for Policy Futures, Faculty of Humanities and Social Sciences, the University of Queensland, Brisbane 4072, Australia.

*Correspondence to: yongping.wei@uq.edu.au

Supplementary Information

A. Supplementary methods

Agricultural and water technology in ancient China

As one of the few existing ancient civilizations, China has been dominated by agricultural activities for most of its pre-industrial history [1]. These agricultural activities relied on technologies developed by collaborative practices, intuitive experiences and observations of natural phenomena [2], which were well documented as a part of a sufficiently long, traceable history in ancient China. Agricultural and water technology in ancient China was therefore chosen as the case study.

The difference between science and technology is nebulous particularly in the ancient period [3]. In this study, a broad definition of technology that does not purposely distinguish between science and technology was adopted. We defined technology as artefacts (e.g. tools and infrastructures), practices (e.g. specific methods for farming and irrigation), and practical routines that "*fulfil certain human purposes in a specifiable and reproducible way*" [4]. Furthermore, we focused on planting

technologies that mobilize land and water resources. Fishery, forestry, animal grazing and husbandry, which were carried out on a limited scale in ancient China [5], were beyond the scope of this study.

Data collection

Content analysis, a well-tested method to systematically extract unstructured textural information for a wide range of studies [6, 7], was adopted to extract technology information from the data sources. The technology information variables were listed in Table S1. The first three variables provided the basic information on the names of specific technologies, and the time and locations where these technologies were invented, developed, or implemented. The final variable was designed to establish connections among technologies, which were based on the evolutionary information between technologies as described in encyclopedias. Two technologies were connected if one evolved based on the other, or where one has influenced/inspired the development of the other. Some examples of connections between technologies (underlined) include: Leisi is a primitive agricultural tool, upon which shovels, sickles and plows were developed; emergence of shovels marked the beginning of cultivation practices; and iron tools for agricultural development were facilitated by iron-making techniques.

Table S1 Technology information variables for content analysis

Technology information variables	
Technology name ("what")	What was the technology?
Time ("when")	When was the technology invented, developed or implemented?
Location ("where")	Where was the technology invented, developed or implemented?
Connection ("how")	How were the technologies connected?

Establishment of the technology network

The agricultural and water technologies extracted from the selected encyclopedias were firstly categorized based on the Chinese Classified Thesaurus (CCT), which is currently the most comprehensive linguistic system used for classification of words in Chinese [8]. As adapted in a previous study [9], a hierarchical structure was established for different technologies based on their

functionalities (Table S2). The classification was mutually exclusive, which meant that one technology can be classified into only one group at any level.

A square matrix was then built to store the connection information between any two technologies to establish a technology network. Each technology was represented by a node, and the influential relationships were represented by edges in the technology network. The technology network was developed based on the following principles: (a) Cumulativeness: all technologies from previous periods were accumulated unless there were specific mentions that they were abandoned, as new technologies were very often adopted to complement existing ones [10]; (b) Interdependency: when connection exists, technologies within the same historical period were assumed to be inter-influencing with each other, i.e. two-directional connections, as inevitable knowledge exchanges among technology users [11]; (c) Uni-directionality: As time is irreversible, technology inheritance can only exist from earlier periods to the later one, but not vice versa.

Both the technology information and connections were cross-checked by two independent coders during the manual coding process to eliminate potential perceptual biases. The Krippendorff's alpha [12] was used to determine the degree of disagreement among the coders, which was kept above 80% as recommended by Poindexter and McCombs [13].

Table S2 Classification system of Agricultural and water technology in ancient China (Retrieved and modified from [9])

Level 2	Level 3	Level 4	
1 Agricultural theory	11 Fertilisation science	111	Organic fertiliser
		112	Green manure
		113	Selection and management of fertilisers
		114	Composting of fertilisers
	12 Soil science	121	Soil formation
		122	Soil biology
		123	Soil classification

				124	Soil fertility
				125	Soil geography and surveying
				126	Regional concept of soil
		13	Agricultural Meteorology	131	Observation of atmospheric elements
				132	Agricultural sub-climates
				133	Observation and forecasting equipment
				134	Observation records
				135	Weather forecasting
		14	Agricultural Biology	141	Agricultural bio-ecology
		15	Farmland management	151	Farmland management practices
2	Agricultural engineering	21	Power sources	211	Manpower/animal power
		22	Tools	221	Primitive tools
				222	Furrowing tools
				223	Seeding tools
				224	Cultivation tools
				225	Harvesting tools
				226	Product processing tools
				227	Irrigation tools
				228	Tools for crop protection
				229	Tools for time keeping
		23	Irrigation infrastructures	231	Irrigation management
				232	Irrigation and drainage methods
				233	Irrigation and drainage engineering
3	Agricultural	31		311	Crop cultivation techniques

practices	Crop cultivation	312	Crop cultivation on artificial land
	32 Crop resource management	321	Crop adoption technique
		322	Germplasm resource
	33 Breeding	331	Productive variety selection
		332	Resistant variety selection and breeding
		333	Breeding technique selection
		334	Fine variety breeding
	34 Furrowing	341	Conventional furrowing
		342	Furrowing in different natural zones
		343	Furrowing in different soil types
		344	Furrowing routines
	35 Planting, sowing	351	Pre-sowing treatment
		352	Sowing technique
		353	Planting technique
	36 Field cultivation	361	Growth management
		362	Cultivating, weeding and fertilizing
		363	Soil treatment
	37 Harvest and storage	371	Harvesting techniques
4 Agricultural protection	41 Natural disaster prevention	411	Droughts
		412	Frost
	42 Bio-physical protection	421	Integrated treatment
		422	Seed treatment
		423	Farming control treatment
		424	Physical treatment
		425	Biological control

	43 Chemical protection	431	Botanical pesticide
		432	Mineral pesticide
5 Agricultural crops	51 Food crop	/	/
	52 Cash crop	/	/

<u>Calculating network features</u>

The network measures calculated for each technology include: degree, closeness, betweenness, and clustering coefficient values, which were embedded functions in the social network analysis software UCINET (https://sites.google.com/site/ucinetsoftware/home):

For any node d (as individual technology) in the network (equation (1) to (4)):

Degree = Sum of no. adjacent edges connected to d; (1)

Closeness = 1/ Sum of the shortest path of d to/from all other nodes (i)

= 1 / Σ shortest distance between (d,i), where i ≠ d; (2)

Betweenness = No. of times d stands between the shortest path of two others (i, j) ;

= Σ shortest distance among (i, d, j) / shortest distance between (i, j), where i ≠ d, j ≠ d, i ≠ j; (3)

Clustering coefficient = Ratio between direct connections and all possible connections to a technology

= adjacent edges connections to d / Σ possible edge connections to d. (4)

In order to ensure comparability among different network measures, each of Degree, Closeness, Betweenness, and Clustering coefficient (raw values) for each technology are normalised using equation (5):

$$Normalised\ network\ measure = \frac{(raw\ measure\ value - min.\ raw\ value)}{(max.raw\ value - min.raw\ value)} \qquad (5)$$

The above values were calculated for each d (each technology in the technology network). The centrality feature was calculated as normalised degree value, and the diversity feature was calculated as normalised clustering coefficient value.

For the adaptability and inertia features, normalised degree (D), closeness (C) and betweenness (B) to measure the level of influences of technologies that are newly developed in a period and those that are inherited from previous periods, respectively. Assume a technology system at a historical period (t) with total number of technologies N, and the number of existing technologies K, which are inherited from previous period (t-1) (equation (6) to (7)):

For an individual technology $i \in (N - K)$:

$$\text{Adaptability of } i = D_i^{C_i^{B_i}} \tag{6}$$

For an individual technology $j \in K$:

$$\text{Inertia of } j = (D_{j,t} - D_{j,t-1})^{(C_{j,t} - C_{j,t-1})^{(B_{j,t} - B_{j,t-1})}} \tag{7}$$

This equation combines the effects of the three indicators at micro-scale to differentiate the comprehensive influences from the legacy technology sub-system and that from the innovative technology sub-system within a network. Due to the accumulated nature of a technology system, we measure inertia for the legacy technology sub-system by the changes of influences from previous periods. A power function is chosen to express the different contribution of the three indicators in their comprehensive effect. This is because the importance of degree, closeness and betweenness is ranked in a descending order, based on their functional characteristics in a network [14]. Similarly, the key technological sub-systems are formed by individual technologies with high inertia and/or high adaptability. The technology sub-systems with both low inertia and low adaptability are considered with limited influences on the entire network behaviours.

Ordinary Least Square (OLS) linear regression analysis and piece-wise linear regression were conducted using R (version 3.5.0, on platform x86_64, apple darwin15.6.0) base function "lm" and package "segmented" (https://cran.r-project.org/web/packages/segmented/index.html), which

calculates the least squares fit in linear model using the QR decomposition method and estimates the break points between two linear regression segments using a score-based approach [15].

The sample sizes (n) for each historical period were:

Yellow River Region: n_Neo = 65, n_XSZ = 132, n_CQZG = 265, n_QH = 395, n_WJ = 582, n_ST = 634, n_SY = 786, n_MQ = 962.

Yangtze River Region: n_Neo = 50, n_XSZ = 53, n_CQZG = 157, n_QH = 190, n_WJ = 257, n_ST = 303, n_SY = 476, n_MQ = 689.

Identifying technological sub-systems at the meso-level

For the Centrality-Diversity feature, technological sub-system in each historical period was identified for those with greater than 0.4 normalised centrality and/or diversity values. A threshold of 0.4 was chosen to ensure the key technological sub-system contains technologies that have at least greater than 50% of normalised values that are separated from other technologies with limited central importance.

For the Adaptability-Inertia feature, technological sub-system in each historical period was divided among those that were inherited from previous periods (legacy technologies) and those that were newly developed in the current period (innovative technologies). The key sub-system included those technologies with greater than 0 normalised adaptability and/or inertia. A threshold of 0 was to ensure any technology with change of network measure are included.

B. The evolution of centrality and diversity values of technology in time for both regions

Figure S1 illustrates the distributions of different types of agricultural and water technologies in terms of their centrality (horizontal axis) and diversity (vertical axis) values for the Yellow River Region and the Yangtze River Region in each historical period. It was observed that the primitive plows ("Leisi"), shovels, and hoes from Agricultural Engineering formed the initial key sub-system from the Neolithic to WJ Period, with the tools developed in the Yangtze River Region being more diversified (i.e. more connected to other technologies and thus used for multiple purposes). More technologies used for protecting crops from harmful disasters and insects were developed and were highly centralised in the Yellow River Region since the WJ Period. During the ST Period, Agricultural Practice technologies appeared to have significant impacts on the centrality and diversity, including systematic cultivation of soils that made planted crops more productive, and development of large-scale ponds and canals that made irrigation and drainage more effective in both regions. The key technological sub-systems gradually contained more agricultural practice methods with high centrality and diversity values, especially for the Yangtze River Region.

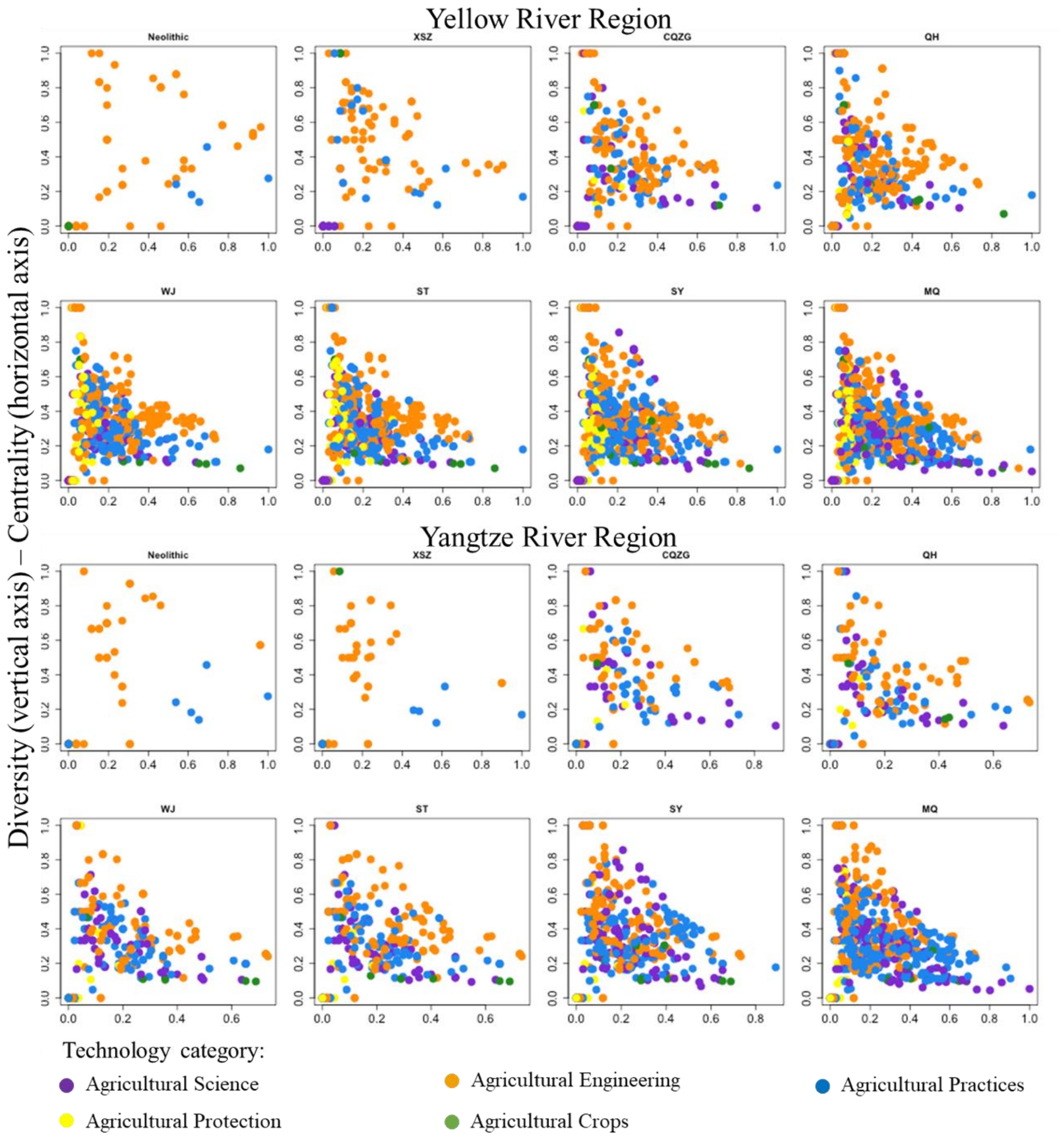

Figure S1 Evolution of relationships between centrality and diversity for both river regions in time

C. The evolution of inertia and adaptability values of technology in time for both regions

Figure S2 illustrates the distributions of different types of agricultural and water technologies in terms of their adaptability (plus symbol) and inertia (dot symbol) values for the Yellow River Region and the Yangtze River Region in each historical period. It was shown that Agricultural Engineering technologies (e.g. tangible tools and irrigation infrastructures) were identified to have high adaptability and inertia during the early Neolithic to WJ Period, especially for the Yellow River Region. In the succeeding periods, increasing numbers of technologies related to farming procedures (Agricultural Practice) and theoretical understandings (Agricultural Theory) were more effective to maintain past knowledge, as well as to stimulate development of innovative technologies for both river regions.

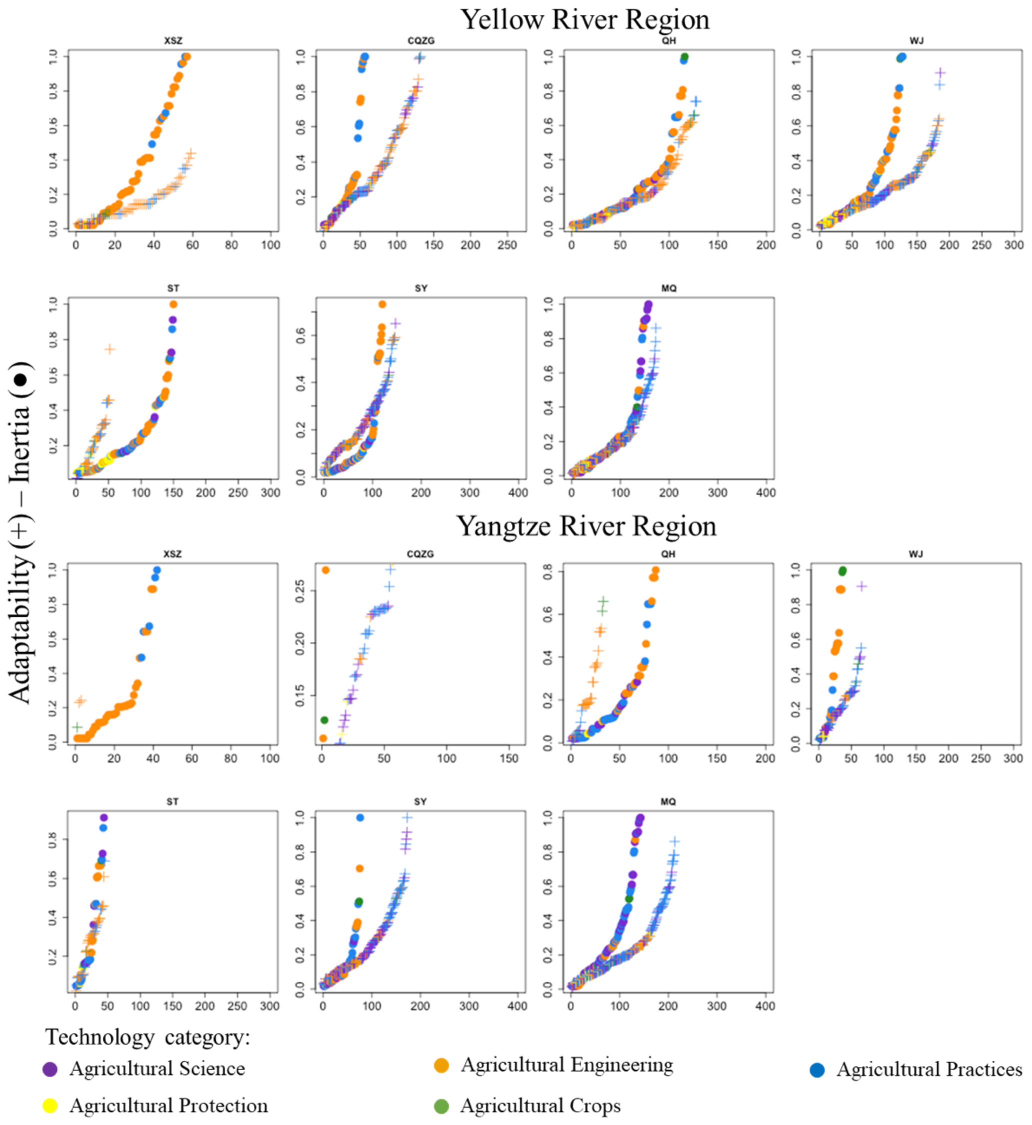

Figure S2 Evolution of relationships between adaptability and inertia for both river regions in time

D. Network matrices and calculated network features for all technologies

A supplementary dataset containing the spatial locations, historical periods, classifications, the original network mactrices and calculated network features for each agricultural and water technologies identified in this study is provided as a separate excel file for full replication of the results presented. Also refer to [9] for more details on the data.

Supplementary References:

1. X. B. Shen, Understanding the evolution of rice technology in China: from traditional agriculture to GM Rice today. *J. Devel. Stud.* **2010**, *46*, 1026-1046.
2. A. Bala, Ed., *Asia, Europe, and the Emergence of Modern Science: Knowledge Crossing Boundaries* (Palgrave Macmillan US, New York)10.1057/9781137031730_1, pp 1-9. **2012**.
3. W. E. Bijker, T. P. Hughes, T. Pinch, D. G. Douglas, *The Social Construction of Technological Systems: New Directions in the Sociology and History of Technology* (MIT press, Cambridge.**1987**.
4. L. D. Anadon *et al.*, Making technological innovation work for sustainable development. *Proceedings of the National Academy of Sciences of the United States of America.* **2016**, *113*, 9682-9690.
5. K. Z. Dong, C. Y. Fan, *The History of Science and Technology in China - The Agriculture Chapter* (Science Publisher, China, pp. 896.**2000**.
6. J. Wei, Y. Wei, A. Western, Evolution of the societal value of water resources for economic development versus environmental sustainability in Australia from 1843 to 2011. *Global Environ. Change.* **2017**, *42*, 82-92.
7. U. H. Graneheim, B. Lundman, Qualitative content analysis in nursing research: concepts, procedures and measures to achieve trustworthiness. *Nurse Education Today.* **2004**, *24*, 105-112.
8. X. Bao, W. Wu, Overview on the Revision Status of Chinese Thesaurus in Recent 40 Years. *Library and Information Service.* **2013**, *57*, 109-113.
9. S. Wu, Y. Wei, B. Head, Y. Zhao, S. Hanna, The development of ancient Chinese agricultural and water technology from 8000 BC to 1911 AD. *Palgrave Communications.* **2019**, *5*, 77.
10. A. Rip, R. Kemp, "Technological change" in Human Choice and Climate Change, S. Rayner, E. L. Malone, Eds. (Battelle Press, Columbus, OH), vol. 2, pp. 327-399. **1998**.
11. E. M. Rogers, *Diffusion of Innovations* (New York: Free Press, ed. 5th).**2003**.
12. K. Krippendorff, *Content Analysis: An Introduction to its Methodology* (Sage.**2004**.
13. P. M. Poindexter, M. E. McCombs, *Research in Mass Communication: A Practical Guide* (Bedford/St. Martin's Boston, MA.**2000**.
14. A.-L. Barabási, R. Albert, Emergence of scaling in random networks. *Science.* **1999**, *286*, 509-512.
15. V. M. R. Muggeo, Testing with a nuisance parameter present only under the alternative: a score-based approach with application to segmented modelling. *JSCS.* **2016**, *86*, 3059-3067.
